# Increase in pediatric recurrent fever evaluations during the first year of the COVID-19 pandemic in North America

**DOI:** 10.3389/fped.2023.1240242

**Published:** 2023-08-03

**Authors:** Leanne M. Mansfield, Sivia K. Lapidus, Samira Nazzar Romero, Lakshmi N. Moorthy, Felice C. Adler-Shohet, Matthew Hollander, Julie Cherian, Marinka Twilt, Geraldina Lionetti, Smriti Mohan, Patricia A. DeLaMora, Karen L. Durrant, Theresa Wampler Muskardin, Mariana Correia Marques, Karen B. Onel, Fatma Dedeoglu, Maria J. Gutierrez, Grant Schulert, the CARRA Autoinflammatory Network Consortium for the CARRA PFAPA/Autoinflammatory Working Group, Shoghik Akoghlanian

**Affiliations:** ^1^ Department of Pediatric Rheumatology, Hospital for Special Surgery, New York, NY, United States; ^2^ Department of Pediatrics, Weill Cornell Medicine, New York, NY, United States; ^3^ Joseph M. Sanzari Children's Hospital at Hackensack University Medical Center and Hackensack Meridian Health, Hackensack, NJ, United States; ^4^ Department of Rheumatology, Nemours Children’s Hospital, Orlando, FL, United States; ^5^ Department of Pediatrics, Rutgers-Robert Wood Johnson Medical School, New Brunswick, NJ, United States; ^6^ Harbor UCLA Medical Center, University of California, Irvine, CA, United States; ^7^ Department of Pediatrics, University of Vermont Larner College of Medicine, Burlington, VT, United States; ^8^ Department of Pediatrics, Stony Brook Children's Hospital, Stony Brook, NY, United States; ^9^ Alberta Children’s Hospital, University of Calgary, Calgary, AB, Canada; ^10^ Department of Pediatrics, University of California San Francisco, Benioff Children's Hospitals, San Francisco, CA, United States; ^11^ Department of Pediatrics, University of Michigan, CS Mott Children’s Hospital, Ann Arbor, MI, United States; ^12^ Department of Pediatrics, New York Medical College, Valhalla, NY, United States; ^13^ Autoinflammatory Alliance, San Francisco, CA, United States; ^14^ National Institute for Arthritis and Musculoskeletal and Skin Diseases, National Institutes of Health, Bethesda, MD, United States; ^15^ Division of Immunology, Department of Medicine, Boston Children’s Hospital, Boston, MA, United States; ^16^ Department of Pediatrics, Harvard Medical School, Boston, MA, United States; ^17^ Department of Pediatrics, Johns Hopkins University School of Medicine, Baltimore, MD, United States; ^18^ Cincinnati Children’s Hospital Medical Center, University of Cincinnati College of Medicine, Cincinnati, OH, United States

**Keywords:** pediatric, fevers, recurrent, COVID-19, rheumatology, CARRA

## Abstract

The impact of the COVID-19 pandemic on new diagnoses of recurrent fevers and autoinflammatory diseases is largely unknown. The Childhood Arthritis and Rheumatology Research Alliance (CARRA) PFAPA/AID Working Group aimed to investigate the impact of the COVID-19 pandemic on the number of pediatric patients evaluated for recurrent fevers and autoinflammatory diseases in North America. The absolute number of new outpatient visits and the proportion of these visits attributed to recurrent fever diagnoses during the pre-pandemic period (1 March 2019–29 February 2020) and the first year of the COVID-19 pandemic (1 March 2020–28 February 2021) were examined. Data were collected from 27 sites in the United States and Canada. Our results showed an increase in the absolute number of new visits for recurrent fever evaluations in 21 of 27 sites during the COVID-19 pandemic compared to the pre-pandemic period. The increase was observed across different geographic regions in North America. Additionally, the proportion of new visits to these centers for recurrent fever in relation to all new patient evaluations was significantly higher during the first year of the pandemic, increasing from 7.8% before the pandemic to 10.9% during the pandemic year (*p* < 0.001). Our findings showed that the first year of the COVID-19 pandemic was associated with a higher number of evaluations by pediatric subspecialists for recurrent fevers. Further research is needed to understand the reasons behind these findings and to explore non-infectious triggers for recurrent fevers in children.

## Introduction

The differential diagnosis for recurrent fevers in the pediatric population is broad. It is challenging to differentiate recurrent fevers related to autoinflammatory disorders and those related to recurrent viral infections, particularly for young children in a childcare or school setting (1). The onset of the COVID-19 pandemic in North America in March 2020 presented an unusual situation with a novel infectious entity, an increased level of concern for fevers, as well as decreased exposure of children to common infections during a time of increased isolation and masking (2–4). Daily life for the vast majority of children with rheumatic diseases was impacted with quarantining (5). The impact of the COVID-19 pandemic on the number of pediatric patients evaluated in North America for recurrent fevers and autoinflammatory diseases is unknown. However, there were reports of increased numbers of children referred for Periodic Fever, Aphthous Stomatitis, Pharyngitis, Cervical Adenitis Syndrome (PFAPA) (6–8).

The Childhood Arthritis and Rheumatology Research Alliance PFAPA/Autoinflammatory Disease Working Group (CARRA PFAPA/AID Working Group) conducts collaborative research to improve care for patients with pediatric rheumatic diseases with a focus on PFAPA and other autoinflammatory diseases. The group's members include pediatric subspecialists in rheumatology, immunology, dermatology, otolaryngology, genetics, and infectious diseases, members of the Autoinflammatory Alliance, and parents of patients impacted by these rare diseases. In the CARRA PFAPA/AID Working Group's meetings at the beginning of the COVID-19 pandemic, increased referrals for recurrent fever evaluations were noted anecdotally among multiple investigators across North America. The group decided to address this issue systematically with a multicenter collaboration to elucidate if it was a true phenomenon. The purpose of this project was to determine the number of new patients evaluated for recurrent fevers by pediatric subspecialists in temporal relation to the COVID-19 pandemic in North America.

## Methods

### Study population and outcomes

**Figure 1:**
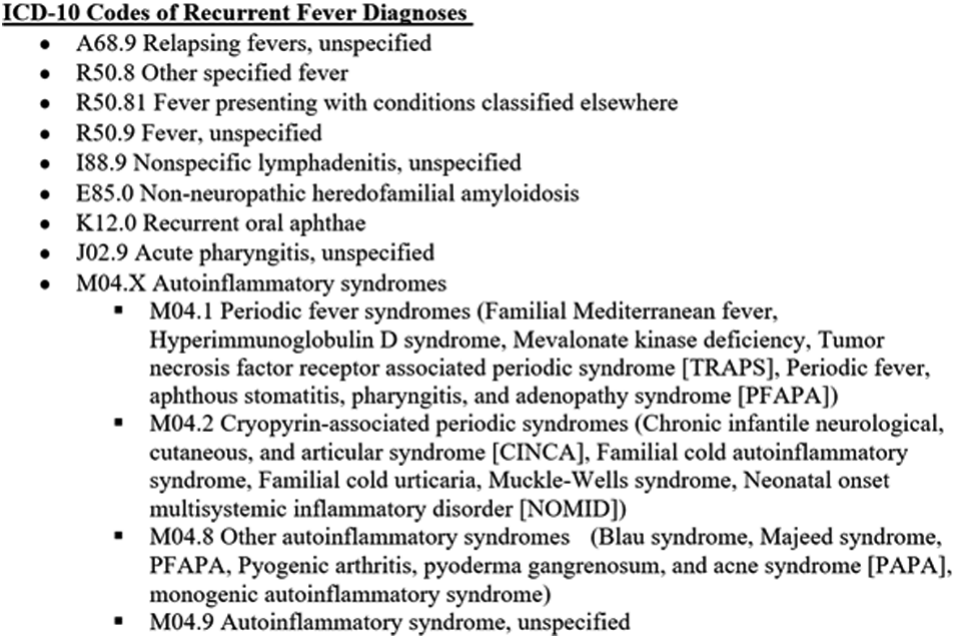
Recurrent fevers and autoinflammatory diseases ICD-10 codes.

The present study included data from 27 academic centers affiliated with the CARRA/AID Working Group and the CARRA Autoinflammatory Network Consortium in the United States of America (U.S.) and Canada. Data was collected individually at the participating centers. A request for data was sent by email to physicians in the CARRA PFAPA/AID Working Group and members of the Consortium. An aggregate of previously determined recurrent fever diagnoses with ICD-10 codes (9) were provided to each participating site (Figure 1). Members were asked to provide total monthly numbers of new outpatients evaluated either in person or by telehealth for recurrent fever diagnoses by pediatric subspecialists from 1 March 2019 to 29 February 2020 (pre-pandemic) and from 1 March 2020 to 28 February 2021 (during the pandemic). Administrative and billing records of participating sites throughout the U.S. and Canada were queried retrospectively. Specialists in pediatric rheumatology, infectious diseases, immunology, and dermatology contributed data. The U.S. sites used ICD-10 codes and Canadian sites used the corresponding names of diagnoses for these data queries. The total monthly numbers of all new patient evaluations at these sites were also requested in order to examine the proportion of new visits attributed to new recurrent fever evaluations. This study was determined to be exempt by the Cincinnati Children's Hospital Institutional Review Board.

### Statistical analysis

Exploratory data analysis was performed to inspect and compare the frequency of children with recurrent fevers at each participating center during the study period. In addition, the proportion of new visits for recurrent fevers relative to all new visits from the pre-pandemic period was compared with the same measure from the first year of the pandemic using a two-sample test of proportions. Our analyses were conducted using STATA version 16 (StataCorp. *Stata Statistical Software: Release 16*. College Station, TX, 2019), and RStudio (RStudio Team, 2022. RStudio: Integrated Development Environment for R, Boston, MA).

## Results

**Figure 2:**
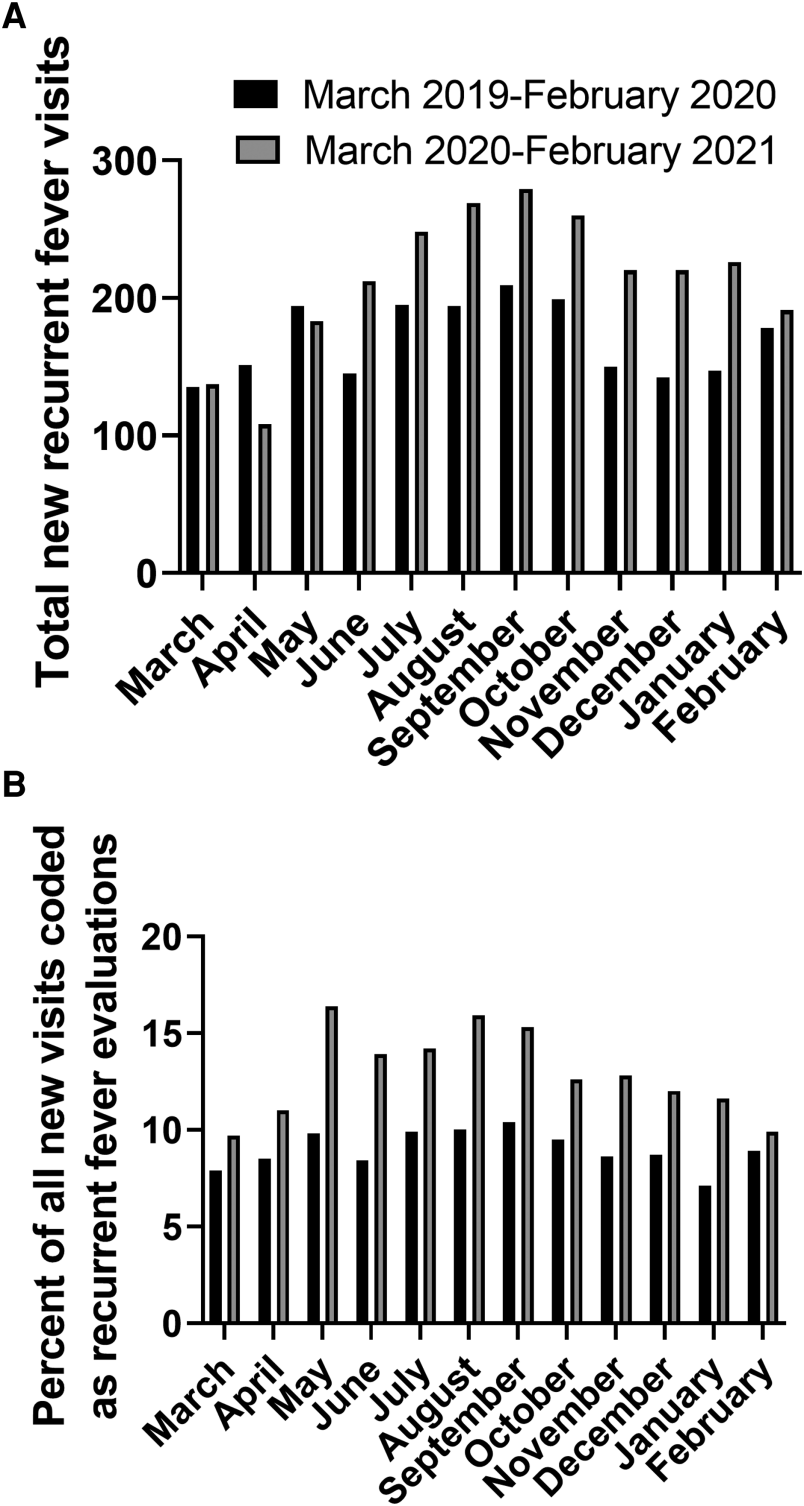
Number of new visits with recurrent fever diagnostic codes (absolute number (**A**) and percentage of all new visits (**B**)).

Data were collected from a total of 27 sites, with 25 sites from 16 states in the U.S. and 2 sites from 2 provinces in Canada. A total of 2,039 new outpatient pediatric visits had received a diagnosis of recurrent fever or autoinflammatory syndrome pre-pandemic, compared with 2,553 during the first year of the pandemic. Temporally, the absolute increase in the number of new clinical visits for evaluation of recurrent fevers began in June of 2020 and continued to be above baseline through February of 2021 (Figure 2A).

**Figure 3:**
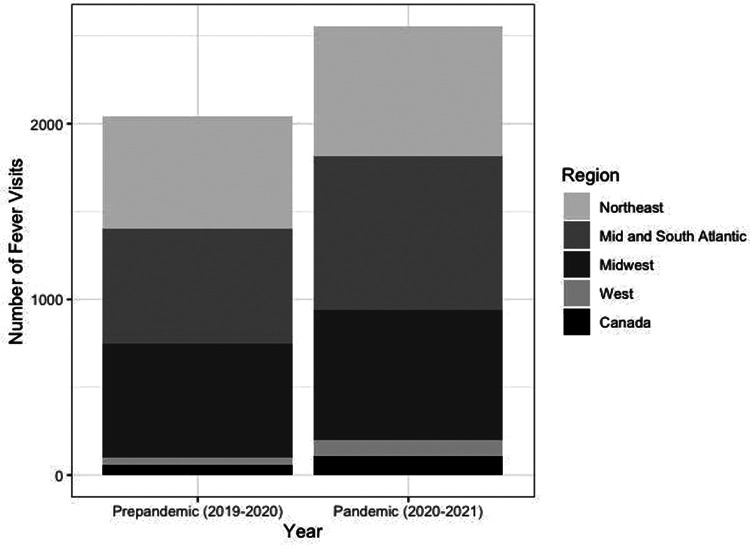
Number of new recurrent fever visits per geographical region pre-pandemic and during the pandemic.

Geographically, there was an absolute increase in the total number of recurrent fever evaluations at the majority of sites (21 of 27 sites) during the first year of the pandemic compared to pre-pandemic. The geographical distribution and increase in recurrent fever new patient evaluations traversed the geographical regions captured in this analysis. The 21 sites with increased new recurrent fever evaluations were distributed across all the participating regions (Canada and the U.S. Northeast, Mid and South Atlantic, Midwest and West) with only 6 centers (2 in the U.S. Northeast, 3 in the Midwest, and 1 in the South Atlantic regions) with decreased number of new recurrent fever evaluations (Figure 3).

For 24 sites, the total number of pediatric new visits (of all diagnoses, including recurrent fevers) during these time periods was obtained. The proportion of new recurrent fever visits in relationship with the total number of new pediatric evaluations at each participating center was calculated. Collectively there were 22,671 new visits pre-pandemic of which 1,766 (7.79%) had a recurrent fever code, compared to 19,788 new visits during the pandemic with 2,157 (10.9%) who received a recurrent fever diagnosis code. Notably, an increased proportion of new recurrent fever visits was noted for each month of the first year after the pandemic onset compared to the year prior (Figure 2B). Overall, the proportion of new visits for recurrent fever evaluations was significantly higher the first year after the start of the pandemic (*p* < 0.0001). There was an increase in pediatric patients evaluated for new recurrent fever diagnoses during the first year of the COVID-19 pandemic compared to the year prior, despite a decrease in the total number of new patients seen during this time.

## Discussion

The CARRA PFAPA/AID Working Group's project is the first collaborative, multidisciplinary effort to assess the number of children evaluated for recurrent fevers in relation to the COVID-19 pandemic in North America. Our data demonstrated a temporal increase in pediatric subspecialty evaluations for new recurrent fever diagnoses during the first year of the COVID-19 pandemic in North America, both in absolute numbers and proportionally in relation with the total new outpatient pediatric encounters in all geographic areas included in this study.

Importantly, early in the pandemic, many subspecialty offices were seeing fewer patients than the preceding year with decreased healthcare utilization (10), repurposing of medical personnel and offices, lockdowns, and social distancing (11). Therefore, it is striking that there were more referrals to subspecialists for recurrent fevers during the first year of the pandemic indicating that the frequency of unprovoked recurrent fevers in children followed an opposite trend. While our findings included but were not limited to PFAPA, they are aligned with previous studies by Geevarghese et al*.* who noted a significant increase in PFAPA diagnoses at their single North American site in 2020 compared to the years prior (8), Ng et al*.* among 957 children at a single center in the United Kingdom (6), and discussed by Sinnappurajar et al. (7) There may be several possible explanations for this phenomenon. We speculate that children with autoinflammatory disorders were recognized earlier because they were having recurrent fevers with minimal infectious exposures from daycare or school, which is consistent with previous reports (6, 12). Additionally, the frequent assessments of temperature due to COVID-19 precautions may also have increased awareness of childhood recurrent fevers.

The increase in new patients evaluated for recurrent fevers also suggests that recurrent fevers in children may be more common than previously thought and perhaps did not prompt early medical attention pre-pandemic as they were attributed to common infections of childhood. For example, Fiorito et al. found a significant decrease in exposure to daycare, travel, and sick contacts in their North American pandemic quarantine group and a shorter, though not statistically significant, time to diagnosis of PFAPA than the pre-pandemic control group (13). Alternatively, we think that the possibility that fevers could have developed after asymptomatic or mild COVID-19 infections cannot be excluded as SARS-CoV2 demonstrated to be a trigger of post-infectious immune dysregulation. This was particularly evident in patients with Multisystem Inflammatory Syndrome in Children (MIS-C) (14), or in susceptible populations (e.g., trisomy 21) who were more prone to develop severe systemic hyperinflammation linked to SARS-CoV2 infections (15).

Finally, it is also possible that physical and/or psychosocial stressors contributed to the emergence of unprovoked fevers in susceptible children as it has been previously described. For instance, Levinsky et al. suggested in a multicenter cohort study that emotional distress was associated with flares of PFAPA during the pandemic (16). Moreover, among the frequently reported triggers for flares of different autoinflammatory syndromes is emotional or physical stress, recognized by 40%–80% of patients with diagnoses of Familial Mediterranean Fever (17–19), TNF-receptor associated periodic syndrome (TRAPS) (20), mevalonate kinase deficiency (MKD) (21), and Behçet's disease (22, 23). Our study could not evaluate physical or emotional stress in children, nor was designed to establish causality. Nonetheless, whether childhood physical and psychosocial stressors may contribute to the triggering of non-infectious fevers in some children is a provocative question that requires additional investigation.

The nature of our data collection for this study poses limitations. First, using ICD-10 codes to ascertain our main outcome allowed us to gather data from many centers, but important demographic and clinical patient characteristics could not be obtained. Second, there is a risk of attrition bias as the use of billing codes at participating sites may vary and the two Canadian sites used diagnoses rather than ICD-10 billing codes to generate the total monthly patient numbers. Third, our data do not extend beyond the first year of the COVID-19 pandemic and the effects of later events such as reopening of schools and vaccinations are not evaluated. Finally, although our study includes data from 27 academic institutions, it does not include all centers where children are evaluated for recurrent fevers in North America and therefore, our conclusions may not be generalizable to regions not assessed.

Nonetheless, strengths of our study include the use of an aggregate of ICD-10 codes (including but not limited to PFAPA), which allowed for a large number of patients evaluated for rare diseases to be studied at many North American pediatric academic centers. While we did not develop or utilize an autoinflammatory disease registry in this CARRA study, its nature supports previously noted benefits of access to registries for generating new data to study these rare diseases and optimize use of global healthcare systems to better care for patients in the future (24, 25). Second, this project demonstrated the CARRA PFAPA/AID Working Group's ability to assemble a large network to answer emerging clinical questions using aggregate patient data. Third, ours is the first known study to highlight the North American experience during the first year of the COVID-19 pandemic regarding pediatric recurrent fever disease evaluations.

In conclusion, our study represents the most extensive multi-institution evaluation of the incidence of new cases of pediatric recurrent fevers during the COVID-19 pandemic in the U.S. and Canada to date. Our work raises thought-provoking questions regarding the potential underlying factors contributing to the observed increase in recurrent unprovoked fevers during a period characterized by reduced infectious exposures in North America. Additional investigations are needed to determine potential causes for our observations, assess if the upward trend in pediatric recurrent fever diagnoses persists in North America, and enhance our knowledge regarding the triggers and mechanisms of non-infectious fevers.

## Data Availability

The original contributions presented in the study are included in the article, further inquiries can be directed to the corresponding author.

## Consortium

CARRA Autoinflammatory Network Consortium: Shoghik Akoghlanian, Cassyanne L. Aguiar, Matthew Basiaga, Joyce Hui-Yuen, Elizabeth A. Kessler, Jamie Lai, Brian E. Nolan, Sheila Nolan, Melissa S. Oliver, Heather O Tory, Sirada Panupattanapong, Maryam Piram, Ann Szymanski, Tiphanie Vogel, and Eveline Wu.
