# Corrigendum: Increase in pediatric recurrent fever evaluations during the first year of the COVID-19 pandemic in North America

**DOI:** 10.3389/fped.2023.1323946

**Published:** 2023-11-06

**Authors:** Leanne M. Mansfield, Sivia K. Lapidus, Samira Nazzar Romero, Lakshmi N. Moorthy, Felice C. Adler-Shohet, Matthew Hollander, Julie Cherian, Marinka Twilt, Geraldina Lionetti, Smriti Mohan, Patricia A. DeLaMora, Karen L. Durrant, Theresa Wampler Muskardin, Mariana Correia Marques, Karen B. Onel, Fatma Dedeoglu, Maria J. Gutierrez, Grant Schulert, the CARRA Autoinflammatory Network Consortium for the CARRA PFAPA/Autoinflammatory Working Group

**Affiliations:** ^1^ Department of Pediatric Rheumatology, Hospital for Special Surgery, New York, United States; ^2^ Department of Pediatrics, Weill Cornell Medicine, Cornell University, New York, United States; ^3^ Joseph M. Sanzari Children’s Hospital, Hackensack, United States; ^4^ Department of Rheumatology, Nemours Children’s Hospital, Orlando, United States; ^5^ Department of Pediatrics, Robert Wood Johnson Medical School, Rutgers, The State University of New Jersey, New Brunswick, United States; ^6^ Harbor UCLA Medical Center, Los Angeles, United States; ^7^ Department of Pediatrics, Larner College of Medicine, University of Vermont, Burlington, United States; ^8^ Department of Pediatrics, Stony Brook Children’s Hospital, Stony Brook, United States; ^9^ Alberta Children’s Hospital, Calgary, Canada; ^10^ Department of Pediatrics, School of Medicine, University of California San Francisco, San Francisco, United States; ^11^ Department of Pediatrics, C. S. Mott Children’s Hospital, Ann Arbor, United States; ^12^ Department of Pediatrics, School of Medicine, New York Medical College, Valhalla, United States; ^13^ Autoinflammatory Alliance, San Francisco, United States; ^14^ National Institute of Arthritis and Musculoskeletal and Skin Diseases (NIH), Bethesda, United States; ^15^ Division of Immunology, Boston Children’s Hospital, Harvard Medical School, Boston, United States; ^16^ Department of Pediatrics, Harvard Medical School, Boston, United States; ^17^ Department of Pediatrics, School of Medicine, Johns Hopkins University, Baltimore, United States; ^18^ Cincinnati Children’s Hospital Medical Center, University of Cincinnati College of Medicine, Cincinnati, OH, United States

**Keywords:** pediatric, fevers, recurrent, COVID-19, rheumatology, CARRA

A Corrigendum on Increase in pediatric recurrent fever evaluations during the first year of the COVID-19 pandemic in North America By Mansfield LM, Lapidus SK, Romero SN, Moorthy LN, Adler-Shohet FC, Hollander M, et al. (2023) Front. Pediatr. 11:1240242. doi: 10.3389/fped.2023.1240242

**Incorrect Author Name**

In the published article, an author name was incorrectly written as “Maria J. Guttierez”. The correct spelling is “Maria J. Gutierrez”.

The authors apologize for this error and state that this does not change the scientific conclusions of the article in any way.

